# Organic and Conventional Bean Pesticides in Development of Autochthonous *Trichoderma* Strains

**DOI:** 10.3390/jof8060603

**Published:** 2022-06-03

**Authors:** Sara Mayo-Prieto, Alessandra Squarzoni, Guzmán Carro-Huerga, Alejandra J. Porteous-Álvarez, Santiago Gutiérrez, Pedro Antonio Casquero

**Affiliations:** 1 Grupo Universitario de Investigación en Ingeniería y Agricultura Sostenible (GUIIAS), Instituto de Medio Ambiente, Recursos Naturales y Biodiversidad, Universidad de León, 24071 León, Spain; gcarh@unileon.es (G.C.-H.); apora@unileon.es (A.J.P.-Á.); 2 Alma Mater Studiorum, Scuola di Agraria e Medicina Veterinaria, Università di Bologna, 40127 Bologna, Italy; alessandra.squarzoni@studio.unibo.it; 3 Grupo Universitario de Investigación en Ingeniería y Agricultura Sostenible (GUIIAS), Área de Microbiología, Escuela de Ingeniería Agraria y Forestal, Universidad de León, Campus de Ponferrada, 24401 Ponferrada, Spain; s.gutierrez@unileon.es

**Keywords:** acaricide, insecticide, fungicide, herbicide, biological control agent

## Abstract

Pesticides of chemical synthesis have mainly been used to control pests, diseases and adventitious plants up until now. However, it has been shown that some pesticides can remain in the soil for long periods of time, thus affecting the development of organisms in the rhizosphere as well as human health, which are two of the most noteworthy side effects. The aim of this research was to analyze the compatibility of autochthonous *Trichoderma* strains with different synthetic fungicides, acaricides, insecticides (including an entomopathogenic fungus) and herbicides. Sulfur encouraged the growth of all autochthonous strains assayed, and the combination *Trichoderma*-*B. bassiana* did not disturb their growth. So, the combination of the autochthonous *Trichoderma* strains with these organic pesticides will be a positive strategy to apply in the field to control pests and some diseases. Conventional pesticides modified the development of all autochthonous *Trichoderma* strains, demonstrating that not only do they affect weeds, fungus or pests but also rhizosphere microorganisms. In conclusion, conventional pesticides indiscriminately used to control pests, diseases and weeds could reduce the development of autochthonous *Trichoderma* strains, especially fungicides and herbicides.

## 1. Introduction

Food consumption must not only be guided by aspects related to the safety of the product but must also be healthy, nutritious and environmentally friendly. Thus, it is necessary to look for production techniques that guarantee consumer safety and environmental sustainability. In this sense, the reduction in chemical measures by using natural production techniques is more highly appreciated by consumers. Until now, pesticides of chemical synthesis have been used mainly to control pests, diseases and adventitious plants. A total of 160,660 tons of fungicides and bactericides were used in the European Union (EU) in 2019 (Spain, France and Italy being the highest consumers, representing 51.56% of the EU); 56,669 tons of insecticides and acaricides (Germany, Turkey and Spain, with 67.74% of the EU); and 128,121 tons of herbicides (France, Spain and Germany, representing 41.72% of the EU) [1]. However, it has been proven that some pesticides can remain in the soil for long periods of time, altering the development of organisms in the rhizosphere and affecting plants, human and animal health. For example, since 2019, the use of the *herbicide Diquat* is no longer approved by the EU due to its high persistence in soil under aerobic conditions. Moreover, it presented a high risk for aquatic organisms or mammals [2]. Another pesticide that has been banned in the EU is Chlorothalonil, based on its very low to moderate persistence in inoculated soils, despite its low risk to soil macro- or microorganisms and its demonstrated efficacy against a broad spectrum of fungal pathogens (EFSA, 2018). Another example of a pesticide is Chlorpyrifos, which has been used as a broad-spectrum organophosphorus insecticide to control insects of the order Hemiptera, such as aphids, larvae of Coleoptera, etc. However, its use has been prohibited because it and some of its derivatives, e.g., chlorpyrifos-methyl-13, have been related to fetus damage caused during neurological development [3]. Mancozeb—used for the control of diseases, such as mildew, *Botrytis*, rust infections, etc., in different plants—has been prohibited because it presents a high risk to birds, mammals, non-target arthropods and soil macroorganisms [4]. All these pesticides have been banned in the EU. However, their use is allowed in other countries.

A biological control agent (BCA) is an organism, other than a human being, that is able to control a phytopathogen or weed or at least reduce their action. They can be bacteria, fungi, viruses, nematodes, mites or insects and have increasingly drawn interest as promising alternatives or as a supplementary way to reduce the use of synthetic pesticides and to ensure environmental and food safety. They can be used as a sustainable strategy in organic farms where pesticides are banned and show a high efficacy against resistant pathogens [5]. Spain is among the countries where a higher number of phytosanitary products are used within the EU, among which only a very small fraction—0.07% of fungicides and 2.08% of insecticides/acaricides—are of natural (microbiological or botanical) origin [6]. There are only a few studies analyzing the effect of phytosanitary products in the development of BCAs, such as *Trichoderma*, which is an Ascomycete frequently isolated from soil, wood, bark, other fungi and from many other substrates. It also exhibited a high opportunistic potential and great adaptability to a wide range of ecological conditions [7]. Crops and all associated microorganisms have co-evolved and adapted to environmental changes. Both partners (plant and microorganism) have evolved to adapt to the changing environment. These changes have resulted in pathogens, which are able to “damage” the plant, and as a result of this process, the biocontrol agents have become more efficient in the control of diseases [8].

Pesticides are used in agriculture to protect crops against pests, diseases and weeds. However, their use has risen with the intensification of production crops, which have resulted in increasing the waste remaining in the soil and reducing the development of soil-beneficial organisms. A combination of pesticides and BCAs improves control against plant pathogens in a more reliable way. Such a combination of *T. virens* and thiophanate-methyl was found to be compatible and more effective than either treatment alone against *Fusarium* spp. in field trials of dry bean production [9]. In another research work, a combined application of *Trichoderma* spp. with a low dose of fluazinam was more effective in controlling *Rosellinia necatrix* Berl. ex Prill. in avocado than either treatment alone [10]. In another work, *Trichoderma* isolates were not effective against *Fusarium oxysporum* Schltdl. and *Acremonium strictum* W. Gams in in vitro conditions, but combining them with a low dose of the fungicide tolclofos-methyl was superior to the fungicide only [11].

In a previous study, we isolated some *Trichoderma* spp. from soil and bean plants at the Protected Geographical Indication (PGI), “Alubia La Bañeza–León” area, where beans were a part of the crop rotation strategy, and evaluated their biocontrol capacity against pests and diseases [8,12,13,14,15,16]. The aim of this research was to study the compatibility of autochthonous *Trichoderma* strains with synthetic fungicides, acaricides, insecticides and herbicides, evaluating their influence on the development of autochthonous *Trichoderma* strains.

## 2. Materials and Methods

### 2.1. Trichoderma Strains

The present study was conducted with autochthonous *Trichoderma* strains collected at PGI “Alubia de La Bañeza León” (European Commission Regulation. Number 256/2010 published on 26 March 2010, Official Journal of the European Journal L0880/17) and stored in the collections “Pathogens and Antagonists” of the Laboratory Diagnosis of Pests and Diseases (University of León, León, Spain) and “Pathogens and Antagonists” of the Research Group of Engineering and Sustainable Agriculture (GUIIAS, University of León, León, Spain) (Table 1). These strains were selected after in vitro and in vivo biocontrol studies in bean crop [8,17,18].

### 2.2. Pesticides

Three acaricides, four insecticides (including an insect entomopathogenic fungus), seven fungicides and three herbicides were tested in this study to evaluate their suitability for application in bean crops (Table 2).

### 2.3. In Vitro Evaluation

**Figure 1:**
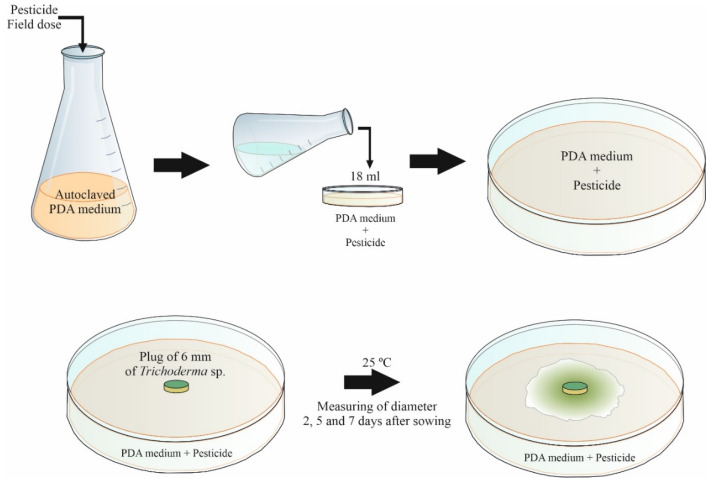
Evaluation of the effect of pesticides in the development of autochthonous *Trichoderma* strains.

Pesticides (Table 2) were incorporated into a potato-dextrose-agar medium (PDA, Merck KGaA, Darmstadt, Germany). Media were sterilized (121 °C, 20 min), the pesticides were added at the recommended field doses and distributed in 90 mm diameter Petri plates (18 mL/plate). Six mm diameter plugs collected from the edge of growing fungal colonies of each isolate were used to inoculate the plates, which were then incubated in the dark at 25 °C for seven days. Control PDA plates without any pesticide were prepared in the same conditions as above (Figure 1).

The development of each *Trichoderma* strain was measured after two, five and seven days after sowing. Experiments were performed with four replicates.

### 2.4. Statistical Analysis

Kolmogorov–Smirnov’s and Shapiro–Wilk’s tests were used to check the normality of the data for ANOVA and variance homogeneity among the treatments.

The results were compared by two analyses of variance. Firstly, a two-way ANOVA for a completely randomized design was carried out, including the main effects of autochthonous *Trichoderma* strains (T008, T019, T028 and T032) and the groups of pesticides (acaricides, insecticides, fungicides and herbicides). The other two-way ANOVA was performed, including the effects of autochthonous *Trichoderma* strains and all pesticides (Table 2). Analysis of Fisher’s least significant difference (LSD) was performed using IBM SPSS Statistics (IBM SPSS Statistics for Windows, Version 26.0. IBM Corp. Armonk, , NY, USA).

## 3. Results

**Figure 2:**
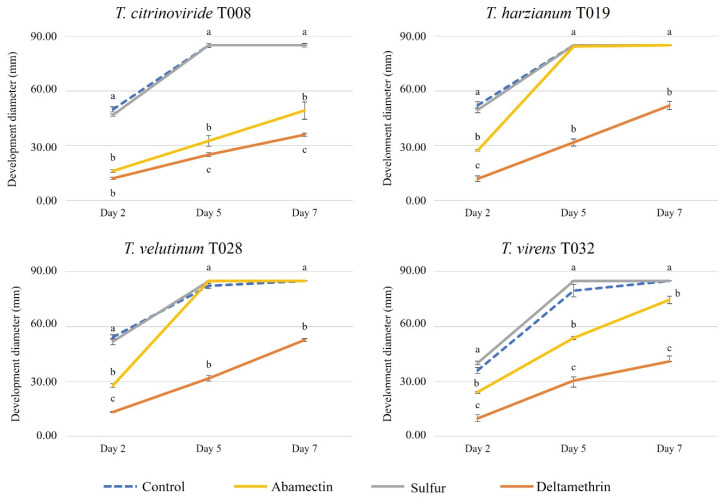
Development of autochthonous *Trichoderma* strains (diameter of growth, mm) in media with synthetic acaricides at 2, 5 and 7 days after inoculation. Blue color: control Petri dish. Yellow color: Abamectin 1.8%. Gray color: Sulfur 80%. Orange color: Deltamethrin 1.5%. The concentrations of each pesticide are specified in Table 2. Upper and lower error bars are represented and indicate standard error of the mean showing the accuracy of the calculations. Different letters indicate significant differences between synthetic acaricides (ANOVA, LSD, *p* < 0.05).

As a result of the Kolmogorov–Smirnov and the Shapiro–Wilk tests, together with the two-way analyses of variance of *Trichoderma* strains and pesticide group on *Trichoderma* development, significant differences were observed among autochthonous *Trichoderma* strains and among the groups of pesticides analyzed but no significant interaction between *Trichoderma* strains and pesticide group (Table 3). The two-way analysis of variance of *Trichoderma* strains and pesticides on Trichoderma development is significant for *Trichoderma* strains, pesticides and the interaction of *Trichoderma* strains x pesticides (Table 4). Therefore, one-way analyses of variance were independently performed for each *Trichoderma* strain and for each group of products (acaricide, insecticides, fungicides and herbicides). The results are presented in Figures 2, 4, 6 and 8.

### 3.1. Acaricides

**Figure 3:**
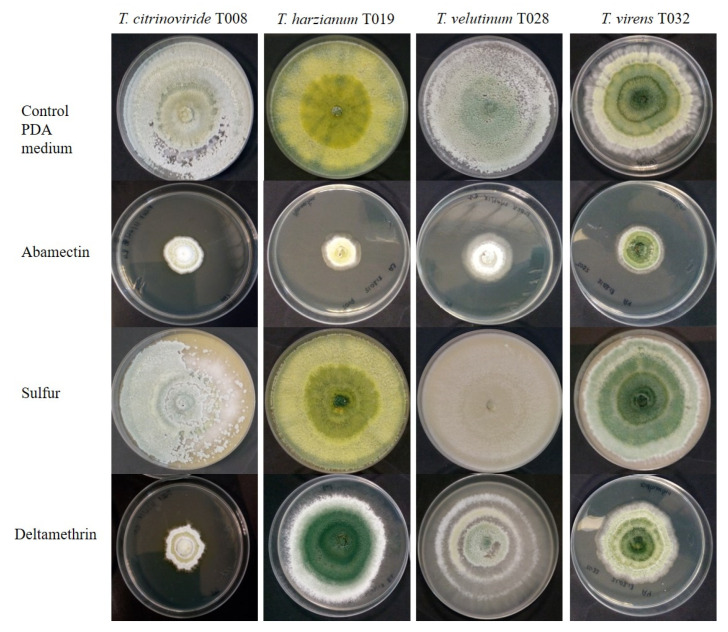
Growth of autochthonous *Trichoderma* strains in medium with synthetic acaricide at day 5 after inoculation.

Regarding the development of *Trichoderma* strains in contact with chemically synthesized acaricides, it was observed that Sulfur did not prevent the development of any tested strain, highlighting *T. virens* where it stimulated the growth compared to the control. In the case of Abamectin, the growth of the different *Trichoderma* strains was lower compared to all the other treatments. However, in the medium with Deltamethrin, all strains grew at a lower level on the second day after inoculation, but this lower development was only observed for *T. virens* and *T. citrinoviride* after 5 and 7 days following inoculation (Figure 2 and Figure 3).

### 3.2. Insecticides

**Figure 4:**
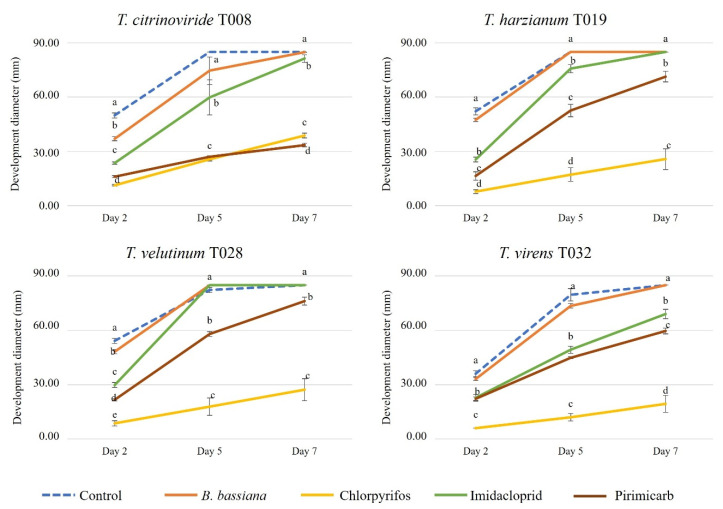
Development of autochthonous *Trichoderma* strains (diameter of growth, mm) in media with synthetic insecticides and the entomopathogenic fungus *Beauveria bassiana* at 2, 5 and 7 days after inoculation. Blue color: control Petri dish. Orange color: *Beauveria bassiana* 22%. Yellow color: Chlorpyrifos 48%. Green color: Imidacloprid 20%. Brown color: Pirimicarb 20%. The concentrations of each pesticide are specified in Table 2. Upper and lower error bars are represented and indicate standard error of the mean showing the accuracy of the calculations. Different letters indicate significant differences between synthetic insecticides and an entomopathogenic fungus (ANOVA, LSD, *p* < 0.05).

**Figure 5:**
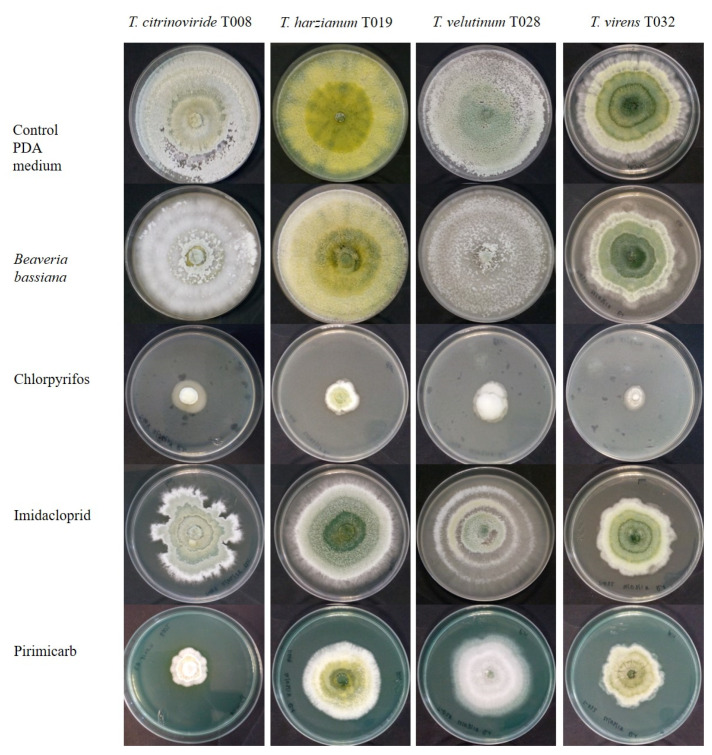
Growth of autochthonous *Trichoderma* strains in medium with synthetic insecticides and the entomopathogenic fungus *Beauveria bassiana* at day 5 after inoculation.

Among the analyzed insecticides was Chlorpyrifos, whose application has been banned by the European Food Safety Authority (EFSA), as it causes problems in children and unborn neurological development. In the current study, we observed that this was the cause of significant reduction in the development of all *Trichoderma* strains, followed by Pirimicarb. *B. bassiana*, while, only significantly modifying the development of *T. citrinoviride* and *T. velutinum* in the first two days after inoculation. Imidacloprid significantly reduced the growth of all *Trichoderma* strains, except *T. velutinum,* when compared to the control (Figure 4 and Figure 5).

### 3.3. Fungicides

**Figure 6:**
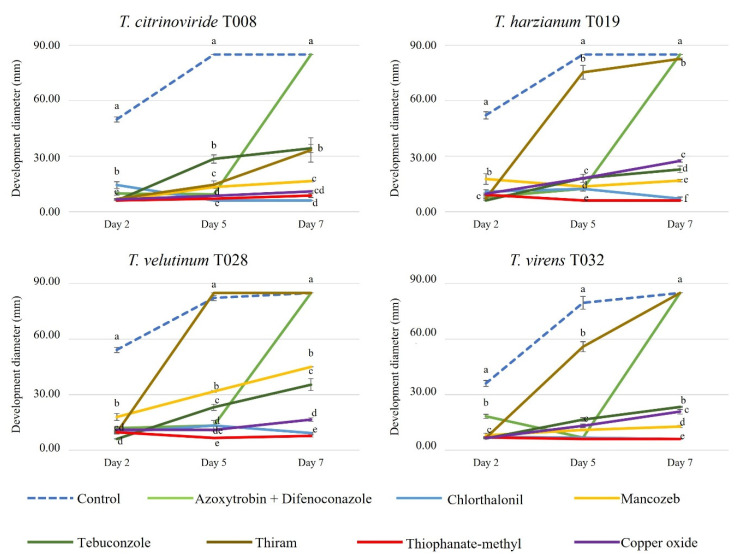
Development of autochthonous *Trichoderma* strains (diameter of growth, mm) in media with synthetic fungicides at 2, 5 and 7 days after inoculation. Blue color: control Petri dish. Light green color: Azoxistrobin 20% + Difenoconazole 12.5%. Blue color: Chlorthalonil 50%. Yellow color: Mancozeb 80%. Dark green color: Tebuconazole 25%. Brown color: Thiram 80%. Red color: Methyl thiophanate 45%. Purple color: Copper 75%. The concentrations of each pesticide are specified in Table 2. Upper and lower error bars are represented and indicate standard error of the mean showing the accuracy of the calculations. Different letters indicate significant differences between synthetic fungicides (ANOVA, LSD, *p* < 0.05).

**Figure 7:**
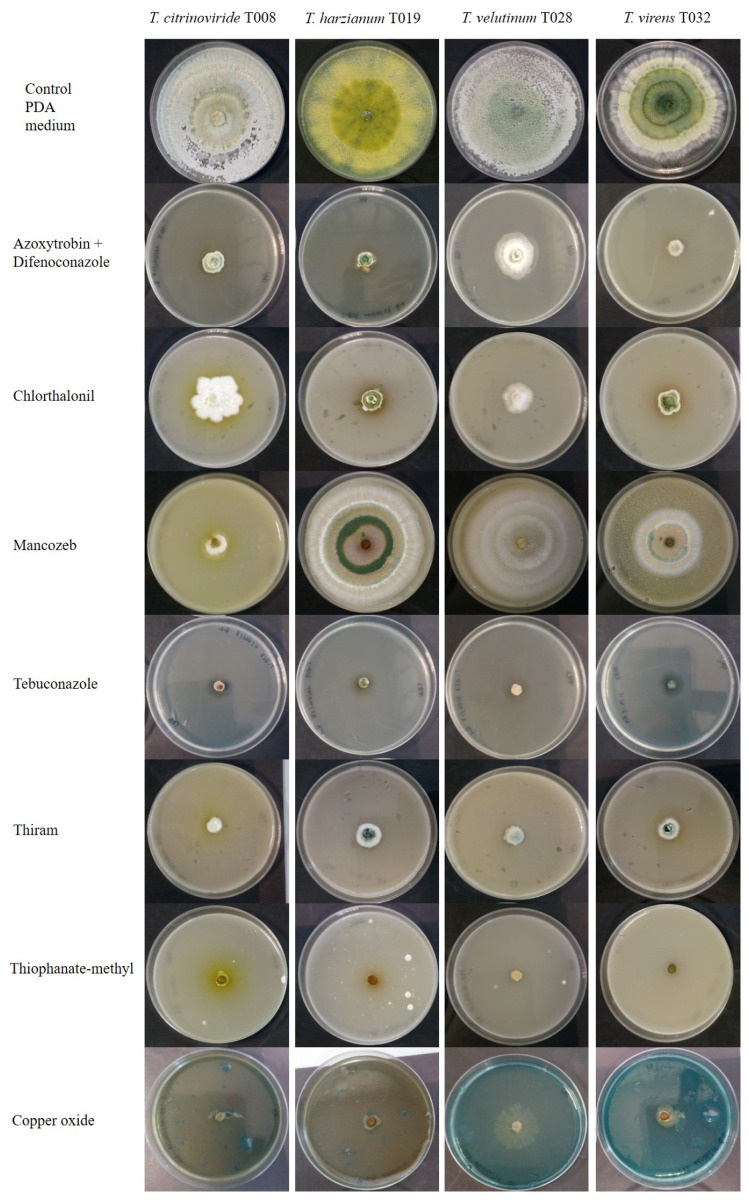
Growth of autochthonous *Trichoderma* strains in medium with synthetic fungicides at day 5 after inoculation.

Mancozeb, Thiophanate-methyl and Thiram are also unauthorized in the EU because they presented a high risk to birds, mammals, non-target arthropods and soil macroorganisms. All autochthonous *Trichoderma* strains assayed were affected by the presence of fungicides in the growth medium (Figure 6 and Figure 7). In the case of Azoxystrobin + Difenoconazole, *Trichoderma* development was affected on the second and fifth day, but on the seventh day, they did not show any differences compared to the control. As for Thiram, its action affected the development of *T. citrinoviride*, which exhibited a lower growth than the control for the evaluation period, but the other strains became similar to the control from the fifth day. Chlorothalonil, Methyl thiophanate, Tebuconazole and Copper drastically reduced the growth of all *Trichoderma* strains analyzed.

### 3.4. Herbicides

**Figure 8:**
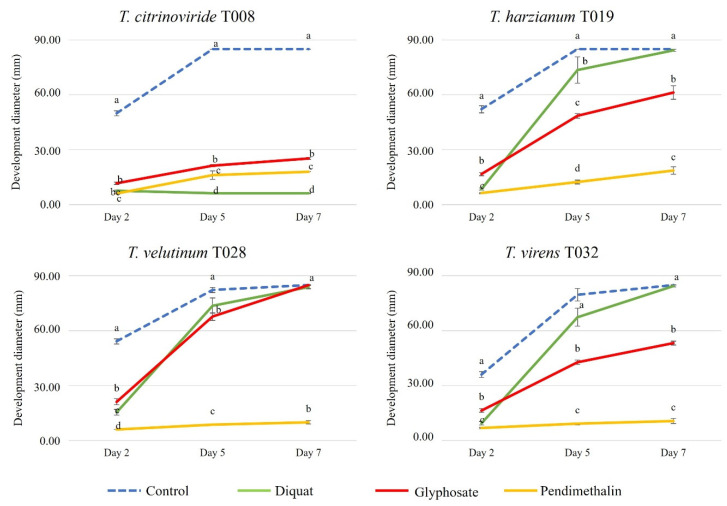
Development of autochthonous *Trichoderma* strains (diameter of growth, mm) in media with synthetic herbicides at 2, 5 and 7 days after inoculation. Blue color: control Petri dish. Green color: Diquat 20%. Red color: Glyphosate 36%. Yellow color: Pendimethalin 33%. The concentrations of each pesticide are specified in Table 2. Upper and lower error bars are represented and indicate standard error of the mean showing the accuracy of the calculations. Different letters indicate significant differences between synthetic herbicides (ANOVA, LSD, *p* < 0.05).

**Figure 9:**
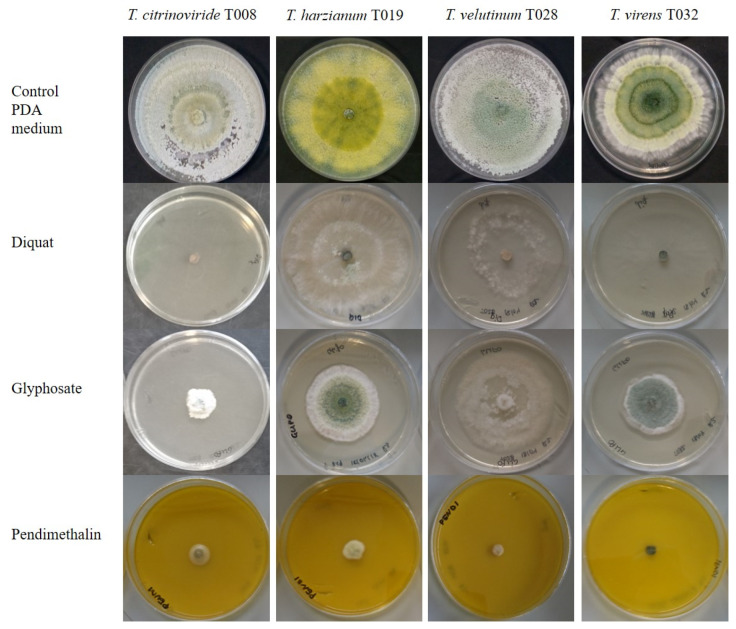
Growth of autochthonous *Trichoderma* strains in medium with synthetic herbicides at day 5 after inoculation.

All herbicides inhibited the development of the autochthonous *Trichoderma* strains but at different levels depending on each strain (Figure 8 and Figure 9). *T. citrinoviride* did not grow or had a weak growth in the presence of all the herbicides analyzed, with Diquat showing the highest level of inhibition. *T. harzianum* and *T. virens* exhibited a lower development than the control, but their growth was not totally inhibited, with Pendimethalin causing the highest inhibition. In fact, this was the only herbicide able to reduce growth of *T. velutinum*, among those tested in the present work.

### 3.5. Trichoderma Development and Groups of Pesticides

**Figure 10:**
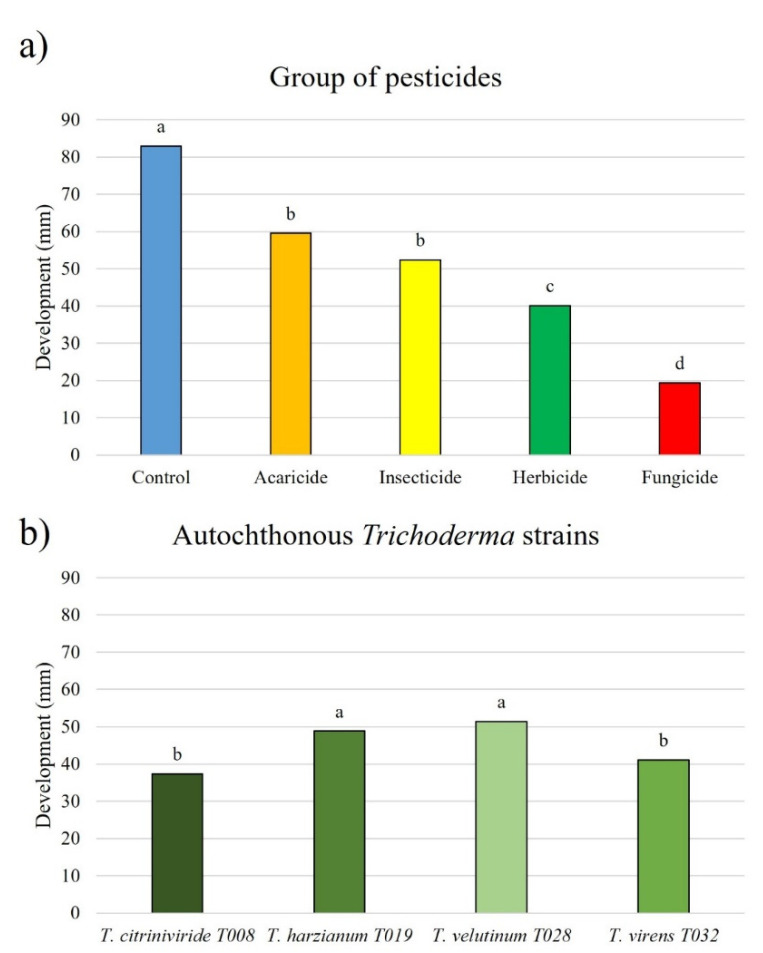
Development of autochthonous *Trichoderma* strains (diameter of growth, mm). (**a**) Analyzing the groups of pesticides. Blue color: PDA medium. Orange color: acaricide. Yellow color: insecticide. Green color: herbicide. Red color: fungicide. (**b**) Autochthonous *Trichoderma* strains (*T. citrinoviride* T008, *T. harzianum* T019, *T. velutinum* T028, *T. virens* T032). Different letters indicate significant differences between synthetic herbicides (ANOVA, LSD, *p* < 0.05).

Analyzing the effect of the pesticide groups on the development of the autochthonous *Trichoderma* strains, it was observed that acaricides and insecticides reduced the development of the *Trichoderma* strains to a lesser extent than the other pesticides. However, herbicides significantly reduced the growth of these BCAs, as well as fungicides (Figure 10a).

Regarding the evaluation of the development of the autochthonous *Trichoderma* strains in the presence of pesticides, *T. harzianum* T019 and *T. velutinum* T028 showed greater development than *T. virens* T032 and *T. citrinoviride* T008, with less growth (Figure 10b).

## 4. Discussion

At present, agriculture is imposing an integrated model of production. The use of chemical compounds is alternated with cultural measures, the application of resistant varieties and the use of agents of biocontrol in order to guarantee environmental sustainability. In a combined treatment of a chemical product and a biological agent, it is suitable to know how the former will affect the development of the latter. Therefore, in this research, the effect of different phytosanitary products on the development of several autochthonous *Trichoderma* strains was studied. There are different research works where the combined use of phytosanitary products and biological control agents produced different responses. This partnership can be positive—by improving the action of both—or negative, by inhibiting growth.

In the case of acaricides, their influence caused different responses in the *Trichoderma* strains. Abamectine, produced by *Streptomyces avermitilis* (ex Burg et al.) Kim and Goodfellow, is firmly fixed to the soil, and it is rapidly degraded by microorganisms [22,23]. The action of this compound was different on each *Trichoderma* strain. Thus, *T. citrinoviride* T008 and *T. virens* T032 had a lower level of growth than the control, which would presumably indicate that they are not able to degrade this compound, but *T. harzianum* T019 and *T. velutinum* T028 growth was not affected.

Deltamethrin is another insecticide–acaricide that eliminates *Euseius* spp., *Amblyseius* spp., *Typhlodromus* spp. and other phytoseids. Its affinity for soils is relatively high, with a half-life of 11–72 days [24,25,26]. This pyrethroid is readily degraded by microorganisms in soil [27,28]. In the present work, the growth of all *Trichoderma* strains was significantly reduced compared to the control.

Sulfur is widespread in agriculture as an acaricide but also as a fertilizer and fungicide. For example, its use is authorized to control mites, such as *Tetranychus urticae* Koch or eriophids, in addition to Powdery mildew. In our research, the growth of all *Trichoderma* strains analyzed was stimulated in presence of this compound. It should be pointed out that *T. virens* development was constantly greater in the presence of this compound than in the control and in all the points analyzed. Previous to this work, it has also been mentioned that Sulfur (2 g/L) significantly increased growth of *T. virens* and *T. harzianum* [29]. Similarly, it was also reported that the use of Sulfur at concentrations up to 500 µg/mL does not affect *T. harzianum* growth [30].

Pesticides are currently being taken off the market in the EU, but in other countries, their application is allowed. Jebakumar et al. [31] studied the compatibility of this pesticide with *T. harzianum* in vitro and in soil, and they observed that Chlorpyriphos could be safely applied with *T. harzianum* for the management of diseases, nematodes or insects. Suseela Bhai and Thomas [32] observed an inhibition of under 8% in *T. harzianum* with this compound in in vitro conditions. In our study, all the strains were inhibited by Chlorpyrifos, with growth diameters less than 30 mm in in vitro conditions.

Pirimicarb is a carbamate with specific activity for the control of aphids. It is systemic, slightly residual and penetrates through the leaves or is absorbed by the roots and translocated through the xylem. Widenfalk et al. [33] observed that pesticides, such as Deltamethrin, Isoproturon or Pirimicarb, induced toxic responses in microbial communities at concentrations that were predicted to be environmentally safe. In another study, *Trichoderma* strains were recorded to efficiently degrade Pirimicarb [34,35]. Unfortunately, in our research, this insecticide inhibited the development of all autochthonous *Trichoderma* strains, with *T. citrinoviride* T008 showing the lowest growth.

Another evaluated insecticide was Imidacloprid. It acts systemically as an antagonist of nicotinic acetylcholine receptors. Some studies focused on its application by foliar spraying, but they are highly toxic to honeybees [36], and residues of this compound were detected in samples from nectar, pollen, plant tissues and soils [37,38]. There are some studies about the combination of this pesticide with BCAs, e.g., combinations of Imidacloprid with entomopathogenic nematodes or with fungi, such as *B. bassiana* or *Metarhizium anisopliae* (Metschn). Sorokīn showed increased insect parasitism [39,40,41]. In the current study, this insecticide produced an inhibition in the development of *T. citrinoviride* T008 and *T virens* T032, on the seventh day. However, the growth of *T. harzianum* T019 and *T. velutinum* T029 was not affected, and they could be good candidates for combination with this insecticide for the treatment of insect pests. Nevertheless, concentrations of imidacloprid must be optimized to avoid its toxicity against honeybees.

*B. bassiana* is an entomopathogenic fungus, which infects the insect by adhering to its cuticle through fungal adhesion proteins [42]. It has been used to control some insects belonging, among others, to the Coleoptera and Lepidoptera orders. Daza et al. [43] observed that a combination of *B. bassiana* and *Trichoderma lignorum* spores was a viable alternative for the control of the leafcutter ant (*Atta cephalotes* L.). In our research, *Trichoderma* development was not inhibited after seven days of incubation, without significant differences with respect to the control plates.

Strobilurin-like fungicides, such as Azoxystrobin, are economically important fungicides that are used against a wide range of fungal-related plant diseases. These compounds can be used alone or in conjunction with BCAs. In a previous research work [44], *Bacillus subtilis* H158, in combination with fungicides of this family (Azoxystrobin, Trifloxystrobin, Pyraclostrobin, etc.), reduced the severity of rice sheath blight caused by *Rhizoctonia solani* J.G. Kühn. Difenoconazole is another triazole fungicide that acts by inhibiting the ergosterol biosynthesis through plant systemic response. A study carried out by Pinto et al. [45] showed that this compound was degraded to levels ranging from 51.3 to 72.1% by *T. viride* and *Fusarium oxysporum* Schltdl., respectively. In the present research, all *Trichoderma* species were affected by azoxystrobin and difenoconazole until the fifth day of growth. However, after the seventh day, their development was not inhibited and matched the control.

Other fungicides used in this research were Chlorothalonil, Mancozeb, Thiophanate-methyl and Thiram. They are fungicides with both preventive and curative activity for the control of a number of diseases in all types of crops. Malandrakis et al. [46] observed that *Fusarium solani* FsK—an isolate used as BCA—was highly insensitive to Thiophanate-methyl and Mancozeb, with an effective concentration as fungicide exceeding 100 mg/mL in in vitro conditions. In another study, some *Trichoderma* strains decreased the conidia production in presence of Mancozeb [47], but in the presence of this compound in the rhizosphere, the soil bacterial communities increased [48]. In the current research, these compounds reduced the growth in all strains. This was an expected result because these are broad-spectrum fungicides, which have been used for a long time to control fungal diseases. With regard to Thiram, only *T. citrinoviride* T008 was inhibited in its development. These results agree with previous data [49], indicating that some *Trichoderma* strains showed low sensitivity to Thiram.

Copper, just like Sulfur, is used as a fertilizer and fungicide. The presence of certain concentrations can cause different responses in the BCA. Banik and Pérez-de-Luque [50] observed that a *T. harzianum* isolate had more sporulation in the presence of Copper. In other research, Lal et al. [51] observed that eggplant seedlings immersed in a solution with oxychloride-dipped Copper and neem cake colonized with *T. harzianum* minimized the wilt incidence compared to some fungicides or BCA used separately. However, in our study, all autochthonous *Trichoderma* strains were affected in their growth by the presence of Copper oxide, which was a contrary result to that observed in other studies where sporulation of some *Trichoderma* strains was not affected by this compound [32,52]. In our research, all strains sporulated after seven days of growth.

Tebuconazole is a broad-spectrum triazole fungicide used worldwide in agriculture for disease control. It has a relatively high soil organic carbon–water binding coefficient and a half-life in soil ranging between 9 and 600 days under aerobic conditions [53,54]. In our study, the growth of autochthonous *Trichoderma* strains was inhibited compared to the control, which would represent a problem for their application as BCA in the presence of this compound. Interestingly, some studies indicate that, after several days following the application of this compound, the soil bacteria population increased significantly at all concentrations assayed compared to the control [55,56,57,58]. This stimulating effect of fungicides could be associated with an increased level of nutrients and energy sources released from the death of fungal hyphae or to a decrease in organisms competing for resources.

While discussing herbicides, we investigated Diquat, a desiccant- , Glyphosate, a systemic non-selective and a persistent, such as Pendimethalin. Unfortunately, *T. citrinoviride* T008 was inhibited by all these compounds. Regarding Diquat, Eberbach and Douglas [59] observed that *Rhizobium* sp. development was significantly retarded by all concentrations of this compound. In our work, *T. harzianum* T019, *T. velutinum* T028 and *T. virens* T032 were not inhibited by this herbicide. Meanwhile, Glyphosate—a non-specific organophosphate pesticide widely applied in weeds—binds to soil particles accumulating in the upper soil layer, often being detected in groundwater and surface water [60,61,62]. Glyphosate can also cause structural changes in local soil microbial communities by inhibiting the growth of soil microorganisms and facilitating the increase in phytopathogenic fungi in the soil [63,64,65]. In our study, *T. velutinum* T028 was not affected by this compound, while *T. harzianum* T019 and *T. virens* T032 were slightly inhibited. Regarding Pendimethalin, which is a dinitroaniline herbicide with residual activity that persists for 3–4 months, some previous research found that, when applied with *Trichoderma*, neither Pendimethalin nor any of the antagonists showed any mycelial radial growth inhibition in the presence of the herbicide at field doses [49,66]. However, in our study, all *Trichoderma* strains were inhibited on all analyzing days, except *T. velutinum* T028, which was only affected by this herbicide.

## 5. Conclusions

Fungicides, acaricides, insecticides (including the entomopathogenic fungus *B. bassiana)* and herbicides were checked in autochthonous *Trichoderma* strains. Even when all pesticides affected *Trichoderma* development, Sulfur encouraged the growth of all autochthonous strains assayed, and the combination *Trichoderma*-*B. bassiana* did not disturb their growth. So, the combination of the autochthonous *Trichoderma* strains with these compounds will be a positive strategy to apply in the field and for controlling pests and some diseases.

As expected, all the tested fungicides (except Azoxystrobin + Difenoconazole) inhibited the development of all autochthonous *Trichoderma* strains. However, all herbicides inhibited the development of all autochthonous *Trichoderma* strains, demonstrating that an excessive use of these pesticides not only affects weeds but also the microorganisms that live the rhizosphere.

In short, it would be necessary to control the use of conventional pesticides, considering that, in general, they could reduce the development of autochthonous *Trichoderma* strains, especially fungicides and herbicides.

## Figures and Tables

**Table 1:** Autochthonous *Trichoderma* strains used in this study.

Isolate ^(1)^	Culture Collection ^(2)^	Species	Crop	Type Sample	Localization
T008	PAULET27	*T. citrinoviride*	Bean	Selected seed	Fresno de la Vega (León)
T019	PAULET38	*T. harzianum*	Bean	Selected seed	Carrizo de la Ribera (León)
T028	IASULE2	*T. velutinum*	Bean	Soil	Villaobispo de Otero (León)
T032	IASULE6	*T. virens*	Wheat	Soil	Cebrones del Río (León)

^(1)^ [8,17]. ^(2)^ All “PAULE” strains are in “Pathogens and Antagonists” collection of the Laboratory Diagnosis of Pests and Diseases (PALDPD), University of León, León, Spain; All IASULE strains are in the “Pathogens and Antagonists” collection of the Research Group of Engineering and Sustainable Agriculture Collection, University of León, León, Spain.

**Table 2:** Characteristics of phytosanitary products used.

Active Ingredient (%)	Mode of Action	Chemical Class	Recommended Field Dose	Observations
**Acaricide**				
Abamectin 1.8% weight/volume (*w*/*v*)	Contact and ingestion	Pentacyclone	80–100 mL/hL	Permanence in soil between 2 weeks and 2 months.
				It is fixed to the ground and is considered immobile on it.
Deltamethrin 1.5% *w*/*v*	Contact and ingestion	Synthetic pyrethroid	50–83 mL/hL	Its activity is reduced with temperatures above 35 °C.
				Non-phytotoxic.
Sulfur 80% *w*/*v*	Direct and remote contact by the gaseous compounds produced	-	250 g/hL	Dose are reduced with high temperature and environmental dryness. Additionally, it had fungicide action.
**Insecticide**				
*Beauveria bassiana* 22% (4.4 × 10^10^ viable spores/g)	Parasitizing the host insect from egg to adult	Fungus: Phylum Deuteromycota	62.5–125 g/hL	It is an entomopathogenic class of insects.
Chlorpyrifos 48 % *w*/*v* ^(1)^	Ingestion, inhalation and contact	Organophosphate	150–200 mL/hL *	It degrades slowly in the soil, with a half-life at 25 °C of 92 to 341 days in acid soils, and from 11 to 200 days in alkaline soils.
Imidacloprid 20% *w*/*v*	Contact and ingestion	Neonicotinoid	50–75 mL/hL	Its residual effect varies between 15 and 21 days in the leaf and 45 and 65 days in the soil, increasing up to 165 and 247 days in very alkaline soils with low organic matter.
Pirimicarb 20% *w*/*v*	Contact, ingestion and inhalation	Carbamate	100 g/hL	It remains in the soil between 7 and 234 days.
				It is stable at pH 4.
**Fungicide**				
Azoxistrobin 20% + Difenoconazole 12.5 % *w*/*v*	Preventive, curative and eradicator effect	Derived from ß-methoxyacrylic acid (Azoxistrobin) Triazole (Difenoconazole)	100 mL/hL	Systemic, few residuals.
Chlorthalonil 50% *w*/*v* ^(2)^	Contact activity Preventive and eradicating action	Polychlorinated aromatic derived from chlorisophthalic acid	250–300 mL/hL	It has a persistence of 1.5–3 months depending on the moisture content and the soil temperature.
Copper 75% *w*/*v* (Copper oxide)	Preventive effect	-	200 g/hL	It is strongly retained in the most superficial area of the soil, being practically immobile.
Mancozeb 80% *w*/*v* ^(3)^	Preventive activity by contact	Diethyldithiocarbamate	200 g/hL	It has a persistence of 6–15 days in the soil.
Thiophanate-methyl 45% *w*/*v* ^(4)^	Preventive, curative effect	Thiocarbamate	300 mL/hL	Secondary action on mite eggs and nematode.
				It is converted to carbendazyme by photodegradation in the soil. Its persistence is approximately 1 month.
Tebuconazole 25% *w*/*v*	Preventive, curative and eradicator effect	Triazole	40–100 mL/hL	It degrades rapidly, and it does not accumulate in the soil.
Thiram 80% *w*/*v* ^(5)^	Preventive activity by contact	Dimethyldithiocarbamate	200 g/hL	Its persistence depends on the pH, concentration and type of soil, varying between 2 days and 32 weeks.
**Herbicide**				
Diquat 20% *w*/*v* ^(6)^	Post-emergence, desiccant and defoliant, with contact activity and non-selective	Bipyridyl	2 L/ha	Residual activity in the soil is of few days, inactivating quickly and completely.
Glyphosate 36% *w*/*v*	Post-emergence, foliar absorption, non-selective	Glycine	3–6 L/ha	It quickly inactivates in the soil. Its persistence in silty-sandy soils is 19.2 days, being several years in clay soils.
Pendimethalin 33% *w*/*v*	Selective control Pre-emergence or early post-emergence	Dinitroaniline	3–6 L/ha	Residual herbicide acting for 3–4 months.

European Commission—Directorate-General for Health and Food Safety (SANTE): non-renewal of the following active substances: (1) Chlorpyrifos (Reg. (EU) 2020/18) [3]; (2) Chlorothalonil (Reg. (EU) 2019/677) [19]; (3) Mancozeb (Reg. (EU) 2020/2087) [4]; (4) Thiophanate-methyl (Reg. (EU) 2020/1498) [20]; (5) Thiram (Reg. (EU) 2018/1500) [21]; (6) Diquat (Reg. (EU) 2018/1532) [2]. Available online: https://ec.europa.eu/food/plant/pesticides/eu-pesticides-database/active-substances/?event=search.as (accessed on 20 May 2022).

**Table 3:** Mean squares of two-way ANOVA (autochthonous *Trichoderma* strains and group of pesticides) for *Trichoderma* development.

Source of Variation	Df ^1^	Day 2	Day 5	Day 7
Autochthonous *Trichoderma* strains (ATs)	3	691.937 **	3431.557 **	3095.672 **
Group of pesticides (Gp)	4	14,308.351 **	39,632.133 **	26,510.961 **
ATs x Gp	12	141.204	371.228	480.613
Error	296	84.303	470.840	651.822
Total	315			

^1^ Degrees of freedom. ** Significant at *p* < 0.001.

**Table 4:** Mean squares of two-way ANOVA (autochthonous *Trichoderma* strains and all pesticides) for *Trichoderma* development.

Source of Variation	Df ^1^	Day 2	Day 5	Day 7
Autochthonous *Trichoderma* strains (ATs)	3	369.093 **	3337.806 **	2357.338 **
Pesticides (P)	17	4594.332 **	15,945.741 **	16,312.180 **
ATs x P	50	71.787 **	476.623 **	461.477 **
Error	245	9.177	27.498	13.094
Total	315			

^1^ Degrees of freedom. * Significant at *p* < 0.05. ** Significant at *p* < 0.001

## Data Availability

The data presented in this study are available on request from the corresponding author.
